# Influence of intergenotypic competition on multigenerational persistence of abiotic stress resistance transgenes in populations of *Arabidopsis thaliana*

**DOI:** 10.1111/eva.12610

**Published:** 2018-03-05

**Authors:** Patrick J. Bigelow, Wayne Loescher, James F. Hancock, Rebecca Grumet

**Affiliations:** ^1^ Graduate Program in Plant Breeding, Genetics and Biotechnology Michigan State University East Lansing MI USA

**Keywords:** environmental biosafety, fitness effects, gene flow, GMO, introgression, risk assessment

## Abstract

Reducing crop losses due to abiotic stresses is a major target of agricultural biotechnology that will increase with climate change and global population growth. Concerns, however, have been raised about potential ecological impacts if transgenes become established in wild populations and cause increased competitiveness of weedy or invasive species. Potential risks will be a function of transgene movement, population sizes, and fitness effects on the recipient population. While key components influencing gene flow have been extensively investigated, there have been few studies on factors subsequent to transgene movement that can influence persistence and competitiveness. Here, we performed multiyear, multigenerational, assessment to examine fitness effects and persistence of three mechanistically different abiotic stress tolerance genes: *C‐repeat binding factor 3/drought responsive element binding factor 1a (CBF3/DREB1a); Salt overly sensitive 1 (SOS1);* and *Mannose‐6‐phosphate reductase (M6PR)*. Transgenic *Arabidopsis thaliana* overexpressing these genes were grown in pure populations and in competition with wild‐type (WT) parents for six generations spanning a range of field environment conditions. Growth, development, biomass, seed production, and transgene frequency were measured at each generation. Seed planted for each generation was obtained from the previous generation as would occur during establishment of a new genotype in the environment. The three transgenes exhibited different fitness effects and followed different establishment trajectories. In comparison with pure populations, *CBF3* lines exhibited reduced dry weight, seed yield, and viable seed yield, relative to WT background. In contrast, overexpression of *SOS1* and *M6PR* did not significantly impact productivity measures in pure populations. In competition with WT, negative fitness effects were magnified. Transgene frequencies were significantly reduced for *CBF3* and *SOS1* while frequencies of *M6PR* appeared to be subject to genetic drift. These studies demonstrate the importance of fitness effects and intergenotype competition in influencing persistence of transgenes conferring complex traits.

## 1 INTRODUCTION

A major target of crop improvement through agricultural biotechnology is reduction in losses due to abiotic stresses (Liang, Prins, van de Wiel, & Kok, 2014; Mittler & Blumwald, 2010). Concerns, however, have been raised about potential ecological impacts if introduced stress tolerance genes become established in wild populations and cause increased competitiveness of weedy or invasive species (e.g., Ding et al., 2014; Hails & Morley, 2005; Lu & Snow, 2005; Nickson, 2008). While presence of transgenes per se does not pose environmental risk, potential for ecological harm could occur if the transgene causes release from ecological constraints leading to population increase or expansion into new geographic regions (Hails & Morley, 2005; Lu & Snow, 2005; Lu, Yang, & Ellstrand, 2016; Warwick, Beckie, & Hall, 2009).

A prerequisite for ecological impacts via population increase or range expansion is establishment of self‐sustaining populations containing the transgene (Guitierrez, Cantamutto, & Poverene, 2011; Hooftman et al., 2011; Kost, Alexander, Emry, & Mercer, 2015; Mercer et al., 2014). Transgenic plants could become feral and form a self‐sustaining population outside of agricultural plantings, perhaps first along field margins, and later by expansion into natural areas. For example, persistent populations of herbicide‐resistant canola resulting from roadside seed spillage have been observed in Australia and Canada, although persistence was limited when plants moved into natural environments (Beckie & Warwick, 2010; Busi & Powles, 2016). Alternatively, the transgene could enter a wild compatible relative through hybridization and introgression. Evidence of naturally occurring crop‐wild hybrids has long been documented for several crops such as such as lettuce, sunflower, radish, oilseed rape and rice; however, concerns about transgenes have led to heightened awareness about the potential for gene transfer from crops to wild populations (Campbell, Snow, Sweeny, & Ketner, 2009; Ellstrand et al., 2013; Hooftman et al., 2011; Lu et al., 2016). Several experimental systems have studied performance of hybrids between transgenic crops and their weedy relatives [e.g., oilseed rape (Halfhill et al., 2005; Rose et al., 2009), rice (Lu et al., 2016)]. In addition, unintended transfer of transgenes has been documented in distant populations of bentgrass in western USA (Zapiola, Campbell, Butler, & Mallory‐Smith, 2008; Zapiola & Mallory‐Smith, 2017), and introgression of herbicide resistance was observed in wild *Brassica rapa* in natural environments over several years, although gene frequency decreased rapidly over time (Warwick, Legere, Simard, & James, 2008).

The key variables influencing gene movement via hybridization, such as mode of pollination, occurrence of compatible relatives, cropping system, population size, and spatial and temporal proximity, have been extensively studied, and several models have been developed to predict extent of gene flow from transgenic crops into recipient populations (e.g., Baker & Preston, 2003; Colbach, Clermont‐Dauphin, & Meynard, 2001; Weekes et al., 2005). However, as emphasized by Ellstrand and Rieseberg (2016), there have been very few multigenerational field experiments tracking transgene persistence. While the extent of pollen‐mediated gene flow is generally independent of the specific transgene introduced, potential gene establishment, and ultimately ecological impact such as weediness or invasiveness, will depend on biology of the crop and recipient wild relatives, abiotic and biotic characteristics of the recipient environment, and importantly, fitness effects of traits conferred by the transgene and surrounding genomic regions (Grumet, Wolfenbarger, & Ferenczi, 2011; Hails & Morley, 2005; Hartman et al., 2014; Keese, Robold, Myers, Weisman, & Smith, 2014; Thompson et al., 2003; Warwick et al., 2009; Xia et al., 2016).

The first wave of commercialized transgenic crops, dominated by Bt‐mediated insect resistance and herbicide tolerance, generally had minimal effects on fitness*,* except under the selective conditions (insect pressure, herbicide application) for which the transgenic crops were developed (e.g., Beckie et al., 2006; Snow et al., 2003; Warwick et al., 2009). These traits are conferred by genes whose protein product is directly responsible for the desired trait: Bt proteins are toxic to the target insect; herbicide resistance genes encode proteins that prevent binding of the herbicide or otherwise inactivate the herbicide. Thus, these genes and their gene products are largely inert with respect to other cellular functions as evidenced by results of global transcriptome, proteome, and metabolome studies revealing that transgene‐induced differences were smaller than the differences that currently exist among conventionally bred varieties (e.g., Cheng et al., 2008; Coll et al., 2008; Ruebelt et al., 2006). These physiologically simple traits may be contrasted with engineered resistances to abiotic stresses which are likely to have complex effects on gene expression, physiology, and growth responses, and may provide variable selective advantages or disadvantages, depending on the environment. Indeed, studies of clinal gradients and reciprocal transplants have found associations between physiological characteristics that influence response to the environment with adaptation to local environmental conditions (e.g., Baxter et al., 2010; Debieu et al., 2013; Wolfe & Tonsor, 2014), and experiments combining common garden and genomic analyses have revealed potential for a small number of quantitative trait loci or effective alleles to modulate local adaptations (e.g., Agren, Oakley, McKay, Lovell, & Schemske, 2013; Hancock et al., 2011). These observations suggest that a small number of genetic changes can influence fitness, depending on the environment.

Research over the past two decades has identified a wide variety of genes with potential for enhancing abiotic stress tolerance such as chaperone proteins, membrane stabilization proteins, metabolic and detoxification enzymes, stress signaling pathway genes, and transcriptional activators (Bhatnagar‐Mathur, Vadez, & Sharma, 2008; Loescher, Chan, & Grumet, 2011). Given the variety of underlying mechanisms associated with such genes, it is anticipated that not all stress tolerance genes would have equivalent effects on fitness and competitiveness of recipient plants. In addition to the selective advantages that may result from the primary, intended effect of the transgene, it is also possible that variable pleiotropic effects resulting from transgene‐induced physiological modifications may also influence fitness, either positively or negatively (Little, Grumet, & Hancock, 2009; Warwick et al., 2009). In addition, extensive cross talk among stress responses, including both abiotic and biotic stresses, has been widely documented (Krasensky & Jonak, 2012; Mittler & Blumwald, 2010). Indeed, transcriptomic analyses comparing three mechanistically distinct genes [*C‐repeat binding factor 3/drought responsive element binding factor 1a (CBF3/DREB1a); Mannose‐6‐phosphate reductase* (*M6PR*), and *Salt overly sensitive 1* (*SOS1*)] which have been shown to confer salt stress resistance as measured by increased survival, dry matter production, and seed yield in growth chamber studies with transgenic *Arabidopsis thaliana* (e.g., Chan, Bigelow, Loescher, & Grumet, 2012; Gilmour, Sebolt, Salazar, Everard, & Thomashow, 2000; Shi, Lee, Wu, & Zhu, 2003; Zhifang & Loescher, 2003), indicate widespread effects on global gene expression even in the absence of applied stress (Chan, Grumet, & Loescher, 2011; Chan et al., 2012). *CBF3* encodes a transcription factor that upon exposure to abiotic stresses such as cold or dehydration induces expression of a cascading series of cold responsive or drought responsive genes, thus modulating a range of stress and growth responses (Fowler & Thomashow, 2002; Seki et al., 2001). *M6PR* encodes a metabolic enzyme that catalyzes the first committed step in mannitol biosynthesis (Zhifang & Loescher, 2003). Mannitol can act as a compatible solute and osmoprotectant, counterbalancing the osmotic and toxic effects of sodium and chloride ions, and as reactive oxygen quencher, reducing damage caused by free radicals produced under salinity stress or in response to pathogen attack (Chan et al., 2011; Sickler, Edwards, Kiirats, Gao, & Loescher, 2007; Zhifang & Loescher, 2003). The *SOS1* gene encodes a plasma membrane Na+/H+ antiporter that shuttles toxic sodium ions away from the cytoplasm, influencing intracellular ionic balance (Shi et al., 2003).

Results from the growth chamber‐grown plants indicate potential for complex fitness effects that could ultimately influence transgene establishment (Chan et al., 2011, 2012). Several studies have indicated the importance of evaluating fitness based on performance throughout the life cycle, in a range of environments, and under competitive conditions (Agren et al., 2013; Bhatnagar‐Mathur et al., 2008; Hartman et al., 2014; Mercer et al., 2014; Mittler & Blumwald, 2010). Therefore, in these experiments, we sought to examine fitness impacts of overexpression of the three mechanistically different abiotic stress resistance transgenes (*CBF3, M6PR, SOS1*) in *A. thaliana* as observed throughout the life cycle over six generations in variable field environments, and to monitor transgene persistence when in competition with wild‐type (WT) plants as would occur during introgression into natural populations. We hypothesized that the mechanistically different transgenes would manifest different fitness effects, and that relative performance would be influenced by direct competition with WT plants. These experiments add to our understanding of factors that may influence transgene establishment and potential environmental impact in native populations.

## 2 MATERIALS AND METHODS

### 2.1 Transgenic lines

*Arabidopsis thaliana* lines 1‐1 and 7‐6 overexpressing *SOS1* in the Columbia glabrous ecotype (Col(gl)) (Shi et al., 2003) were provided by Huazhong Shi, Texas Technological University. The *CBF3/DREB1a* overexpression lines, A30 and A40, in the Wassilewskija ecotype (WS) (Gilmour et al., 2000) were provided by Michael Thomashow, Michigan State University. *M6PR* overexpression lines M2‐1 and M5‐1 in the Columbia ecotype (Col) were as described in Zhifang and Loescher (2003). All lines represented unique transformation events. Each of the abiotic stress resistance transgenes was expressed constitutively using the *Cauliflower mosaic virus* (*CaMV*) 35S promoter. The lines provided were previously demonstrated to confer salt stress resistance in growth chamber studies (Gilmour et al., 2000; Shi et al., 2003; Zhifang & Loescher, 2003). With the exception of *CBF3* A30, all also conferred resistance to 100 mM salt stress in our growth chamber experiments relative to WT parents as measured by reduced salt injury, greater dry weight, and increased seed yield (Chan et al., 2012). Each line also contains the neomycin phosphotransferase II (*NPTII*) gene for kanamycin resistance as a selectable marker under control of the nopaline synthase (*NOS*) promoter. The WT parents [Col, Col(gl) and WS] are all highly homozygous, rapid‐cycling *A. thaliana* ecotypes. For each transgenic line, the initial seed provided was generation *T*_2_ or *T*_3_, derived by self‐pollination from the original molecularly verified transgenic plant. Plants grown from the seed provided were verified to contain and express the specified transgene by PCR, Southern and Northern blot analysis (Figure S1) and verified to be homozygous by kanamycin screening of progeny seed. Verified plants were used for seed amplification by self‐pollination in the glasshouse to provide sufficient starting material for the first generation of the field experiments.

### 2.2 Field experiment design

The experimental design for the multigenerational field study was adapted from a glasshouse experiment examining long‐term fitness effects of *Arabidopsis* mutations under competitive conditions (Roux, Camilleri, Bérard, & Reboud, 2005). Planting density (2600 seeds/m^2^) [yielding ~180 (±20%) seedlings per 26 × 26 cm tray] was selected to ensure a naturally high level of interplant competition for each population as was previously utilized by Roux et al. (2005). Fourteen replicate intergenotypic competitive populations (one tray = one population = ~180 plants) were established for each transgenic line and background WT mix with a starting allelic frequency of 50% transgenic and 50% WT. This level of replication was chosen to ensure that fitness impacts from transgene expression ≥5% would be detectable after three generations by exceeding the 95% confidence intervals attributable to genetic drift based on theoretical distributions using the following formula, *V*_qt_ = *q*_0_ *p_0_ *(1‐(1‐1/(2*N*_e_))^*t*^) (Falconer & Mackay, 1996). The initial frequencies of transgenic and wild‐type plants at the *t* generation are *q*_0_ and *p*_0_ *,* respectively, and *N*_*e*_ is the effective population size. Due to the highly selfing nature of *Arabidopsis thaliana*, 98%‐99% (Snape & Lawrence, 1971), the effective population size was calculated based on the following formula, Ne = *N*/(1 + (β/(2−β)) (Caballero, 1994) where β is the proportion of selfing (0.98) and *N* is the observed population size (180), for an effective population size of 92. Confidence intervals were derived as the expected mean ± (1.96*(*Vqt*/14)^0.5^) with 14 being the number of replicate populations and the expected mean equal to the starting frequency, 0.5, due to the assumption of no selective pressure.

In addition to the mixed populations, five pure populations of each genotype grown at the same planting density as the mixed populations were included for comparative purposes. A total of 129 populations were grown for each of the six generations [84 mixed populations and 45 pure populations in each season (Table S1)]. To initiate each generation, all seeds were first planted in the glasshouse by direct seeding onto the soil surface of 26 × 26 cm trays with perforated bottoms (L‐HFT NCR, Landmark Plastics Corporation; Akron, OH). The trays were filled with Baccto potting soil (Michigan Peat Company) mixed with 2 g/L Slow release Osmocote Classic^®^ fertilizer (The Scotts Company LLC, Marysville, Ohio), premoistened, and lightly compacted to create a firm seed bed. The number of seeds planted for each population was based upon viability tests of seeds derived from the prior generation to provide enough viable seeds for 180 seedlings. Seeds used for planting were counted using custom made counting plates (20‐gauge galvanized steel plates were drilled with 1.98 mm holes providing a standard number of seeds per hole). The counted seeds were mixed with sterile dry white laboratory sand to improve seed scatter and seeding visibility and stratified at 4°C for 48–60 hr prior to planting. The trays were placed, two per flat, into 27.4 × 53.9 cm glasshouse flats (L‐1020NCRN, Landmark Plastics Corporation) and arranged in a computer generated, completely randomized design. All watering was performed via subsoil irrigation. Temperature was kept between 21–25°C, and supplemental lights were used to provide an 18‐hr light period.

Once the study populations reached the rosette stage (5–6 true leaves), they were transferred to the field at the Michigan State University Horticultural Teaching and Research Center (HRTC; N 42, 40.426′; W 084, 28.948). Field experiments were initiated in June 2008, September 2008, May 2009, September 2009, May 2010, and September 2010 in accordance with the specifications of USDA‐APHIS notifications 08‐065‐104n, 09‐080‐101n, and 10‐070‐119n with regard to contained movement of materials between laboratory, glasshouse, and field; autoclaving and/or disposal of containers; location and security of the field site; containment of plants and seeds during the field season, including isolation distance from native *Arabidopsis* populations and maintenance of an *Arabidopsis*‐free zone surrounding the test plots; removal of plants to prevent dissemination of seeds; harvesting and storage of seeds to assure containment; treatment of residual plant material after removal of flower stalks; treatment of the field location with herbicide; and monitoring of the field location for the volunteers in the following year. The populations were arranged in a computer generated, completely randomized design. A tray‐in‐flat potting method was developed to allowing for subsoil irrigation via trickle hose and provide secure anchoring to the ground (Figure S2a). Plants were grown in the field until approximately 75% of siliques had begun to senesce. The trays were then returned to the glasshouse for final maturation and dry down prior to harvest. This procedure minimized seed loss prior to harvest, assuring seed confinement and increasing accuracy of seed yield measurements.

At harvest, all aboveground plant material was harvested from the tray, transferred to paper bags, and allowed to dry to ambient humidity levels. The dried plant materials were cleaned of seed. Seed derived from each population (pure line or mixed) was maintained separately. Seed viability was tested by percent germination following 48‐ to 60‐hr stratification at 4°C (three batches of 100 seeds were tested for each population at each generation). Calibration counts (relative to the custom plate hole size) were performed for each population at each generation to compensate for possible differences in seed size due to genotypic and/or environmental effects. The number of seed planted for each population in each generation was adjusted as needed for seed size and viability to provide approximately 180 seedlings per population to initiate the subsequent generation.

Climatic data were recorded at the HTRC weather station, located less than 400 m from the field site. The weather station recorded air temperature, precipitation, solar radiation, potential evapotranspiration, and wind speed across all six field growing seasons. Information about mean daily maximum and minimum temperatures and mean daily total solar flux for each of the six seasons is provided in Figure S3.

### 2.3 Developmental and productivity data

Developmental data were collected on all pure populations on a per tray basis as days postsowing when ~75% of the population reached the following developmental stages: germination; two true leaves emerged; rosette formation (5–6 true leaves); bolting; flowering; siliques mature. Time of first bolting and time of first flowering also were recorded. At harvest, all aboveground plant material from each replicate population at each generation, from both pure and mixed populations, was removed and allowed to dry to ambient humidity levels. The dried plant materials were cleaned of seed, and total plant dry weight and seed mass were recorded. Seed viability (percent germination) and seed size were tested as described above for each population in each generation. Relative fitness of transgenic lines was derived from comparison of the mean viable seed yields of the pure transgenic populations to the mean viable seed yields of the corresponding WT background in each season (five replicate pure populations per season).

Seasonal and genotypic effects on development and productivity were analyzed by analysis of variance (repeated‐measures ANOVA) of pure line data [nine lines (WT and two transgenic lines per transgene), six seasons, five replicate populations per line per season]. All statistical calculations and comparisons were performed with the SAS version 9.2 and R version 2.14.0 statistical programs (SAS Institute Inc., Cary, NC and R Foundation for Statistical Computing, Vienna, Austria). Productivity measures of the transgenic lines were compared to their respective WT parents by paired *t* test; mean performance of transgenic lines in each season was compared to the respective WT parent in the corresponding season (*n* = 6 seasons).

### 2.4 Genotyping field grown progeny via kanamycin screening

Transgene frequency was monitored in each replicate population of the mixed populations at each generation by kanamycin screening of progeny seed to determine relative gene frequency of the NPTII selectable marker gene. Sterilized seed were plated via 20‐μl pipette, onto ½ MS media +1% agar containing 100 mg/L kanamycin (with the exception of lines A40 and S1‐1 which were plated onto media containing 75 mg/L kanamycin due to a lower level of resistance than the other transgenic lines). Kanamycin screenings for the mixed populations were performed in triplicate for each population and generation with 100 scored seedlings/replicate plate. Each plate also included an aliquot of seed from the respective WT as negative controls to verify kanamycin effectiveness. WT seedlings showed complete bleaching and did not proceed past the cotyledon stage, while transgenic seedlings grew normally; resistant and susceptible individuals were also easily distinguished from seeds that failed to germinate (Figure S2b).

The overall mean germination rate for all populations was 92.62 ± 0.33%, indicating minimal impact of the sterilization procedure or the presence of kanamycin in the media on seed viability and germination. Median germination percentages of pure populations ranged from 89% to 97% (Figure S4). Continued presence and expression of the transgenes in pure transgenic populations, and absence in WT lines, also were verified by kanamycin resistance screening after each of the six generations.

### 2.5 qPCR (quantitative polymerase chain reaction) verification of transgene frequency

Progeny seed was sterilized as described above and plated via pipette on to ½ MS media +1% agar, and grown to the two‐leaf stage. A set of standards was created by producing mixes of confirmed transgenic or WT seedlings to result in batches of 100 seedlings with 0% transgenic, 10%, 20%, 50%, and 100% transgenic plants. Seedlings were harvested in batches of 100, weighed, and flash frozen in liquid nitrogen. DNA extraction was performed using the Wizard^®^ Genomic DNA Purification kit (Promega Corporation, Madison, WI, USA) according to the manufacturer's instructions. DNA quality was confirmed via gel electrophoresis, and DNA quantity was calculated using the Qubit^®^ fluorometric quantification system (Life Technologies Corporation, Invitrogen™, Grand Island, NY, USA). The qPCR was performed using primers for the NPTII gene on a Stratagene Mx4000 (now Agilent Technologies Inc., Santa Clara, California) (forward 5′‐ CGGCTGCATACGCTTGATC‐3′ and reverse 5′‐GATGCGATGTTTCGCTTGGT‐3′) and SYBR^®^ Green master mix (Life Technologies Corporation, Applied Biosystems^®^, Grand Island, NY, USA). Transgene frequency within the mixed populations was calculated by comparison of each replicates Ct values to the standard curve of Ct values established from the known transgene frequency standards.

## 3 RESULTS

These experiments examined fitness effects and persistence of three mechanistically different abiotic stress tolerance transgenes, *CBF3, M6PR,* and *SOS1*. Populations of *A. thaliana* plants overexpressing these genes (two independent lines per transgene) were grown over six generations, with and without competition from their respective WT parents. Seed for subsequent generations in both pure line and mixed populations was from those produced during the prior generation. The planting density (~180 plants/tray; 2,600 seeds/m^2^), used for both pure and mixed population experiments, was within the range of natural population densities we observed growing in ruderal habitats in the area of East Lansing, Michigan (769–3,254 plants/m^2^). Although *Arabidopsis thaliana* is generally classified as a winter annual, that is, germinating and forming a rosette in the fall and flowering and setting seed the following spring, naturally occurring populations can exhibit spring or fall flowering phenotypes (Griffith, Kim, & Donohue, 2004; Pigliucci & Marlow, 2001). The pure population and intergenotype competition experiments used both spring and fall plantings over 3 years to mimic the growing periods of natural populations of *A. thaliana* observed in Michigan (Griffith et al., 2004; observations made during this study period). These planting periods exposed populations to the range of environmental variations typical for this location.

**Figure 1:**
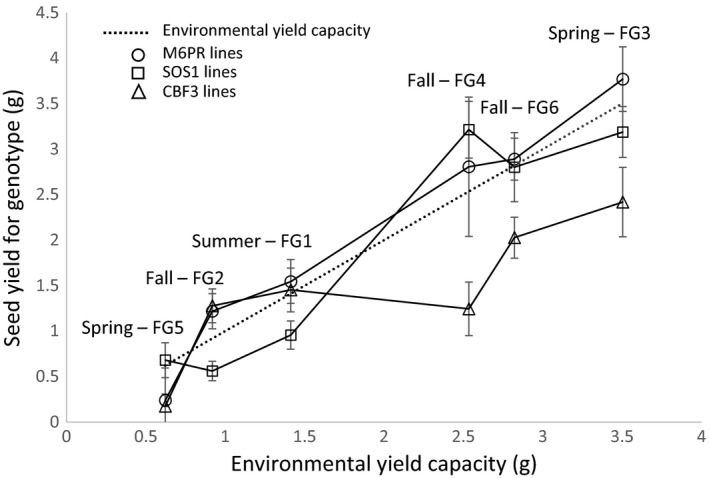
Mean yield of transgenic lines in each season in relation to the environmental yield capacity of that season. The environmental yield capacity (g seed weight) was the mean yield for all pure populations grown in that season [*n* = 45; nine lines (two transgenic and one WT for each of three transgenes) and five populations/line per season]. The CBF3, SOS1, and M6PR values for each season are the mean seed yield (g) of 10 populations ±*SE* (two lines per transgene, five populations per line)

Seasonal differences in environmental factors such as temperature, day length, light intensity, rainfall, and humidity reflect the range of environmental conditions experienced by natural populations (Figure S3). Significant variation in plant development and productivity was observed across the six growing seasons as measured in the pure populations (Tables 1 and S2). For example, plants grown in Fall 2008 (generation 2), which had the lowest average maximum and minimum air temperatures of the six field seasons, were the slowest to reach every developmental stage. Plants grown in generation 5 (Spring 2010), which was marked by very heavy rainfall and frequently saturated soil, produced the least dry matter and seed yield of the six seasons. Mean aboveground dry weight averaged over all genotypes varied 2.5‐fold across the six planting periods (7.45 g/tray–18.72 g/tray); mean seed yield varied fourfold [0.83 g/tray–3.56 g/tray] (Figure 1, Table S2). Seasonal effects did not uniformly affect all populations, indicating significant genotype by environment (GxE) interactions (Figure 1, Table 1).

**Table 1:** Repeated‐measures analysis of variance of seed yield of pure populations grown in six field seasons

Source	Degrees of freedom	Sum of squares	Mean square	*F* value	Probability
Model	53	464.16	8.76	4.21	<.001
Environment (season)	5	298.15	59.63	28.67	<.0001
Genotype	8	82.75	10.31	4.96	<.001
Genotype*Environment	40	83.26	2.08	2.39	<.001
Pooled Error	216	31.32	0.87		

### 3.1 Performance of pure populations

**Figure 2:**
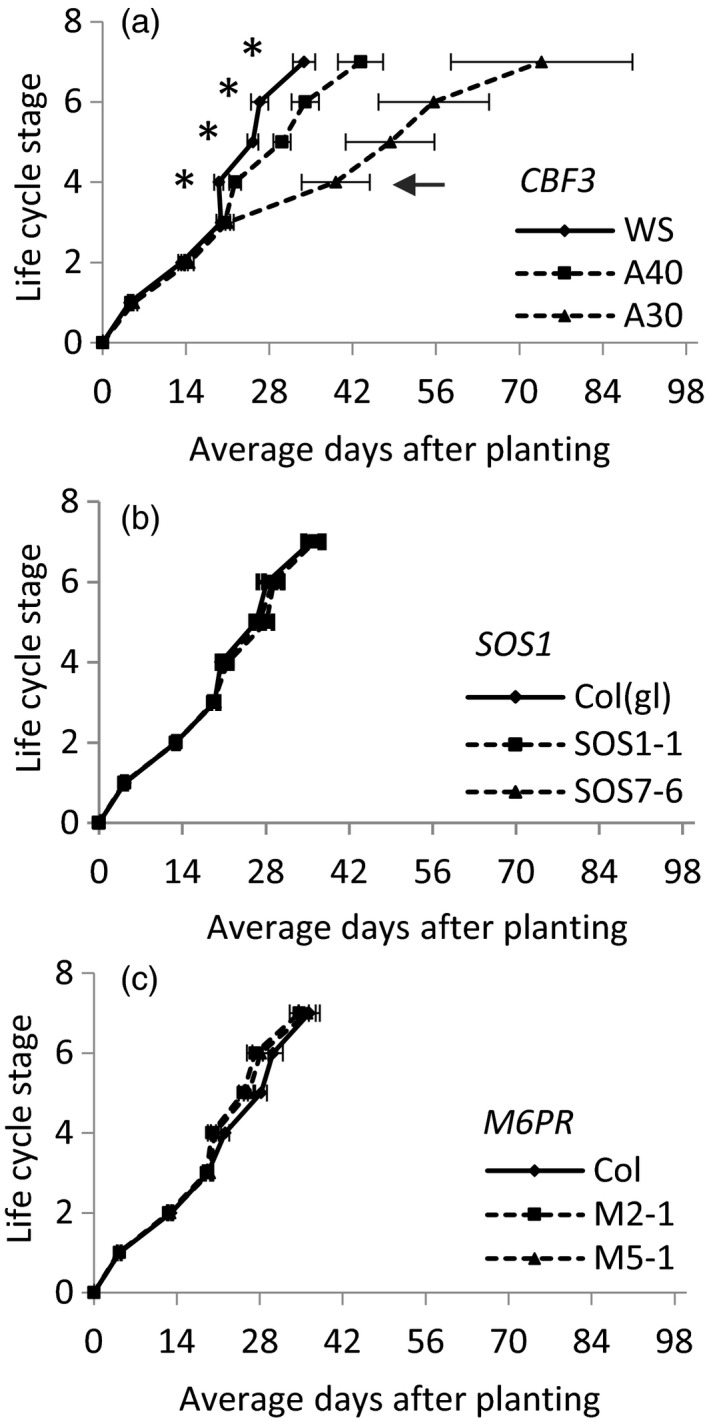
Mean number of days to reach life cycle stages for pure populations of wild‐type (WT) (solid) and transgenic plants (dashed). (a) *CBF3* overexpression lines A40 and A30 and WT WS. (b) *SOS1* lines S1‐1 and S7‐6, WT Col(gl). (c) *M6PR* lines M2‐1 and M5‐1, WT Col. Life cycle stages: first germination (1), 75% of the population with two true leaves (2), 75% with 5–6 true leaves (rosette) (3), first bolting (4), first flowering (5), 75% reaching bolting (6), 75% flowering (7). The transition from vegetative to reproductive growth is indicated by the arrow in panel (a). Values are the mean ± SEM of five replicate populations/line/season averaged over six seasons (in some cases error bars do not exceed width of the data point marker). Values for *CBF3* lines A40 and A30 stages 4–7 are significantly different from WT,* p* < .05 as determined by analysis of variance and Duncan's multiple range test

Performance of the transgenic lines in pure populations over the six growing seasons showed different impacts of the introduced genes on plant development and productivity. *CBF3* lines had delayed reproductive development relative to WT (stages 4–7, *p* < .001, Figure 2a), while *SOS1* had no discernible effect on development (*p* > .8, Figure 2b). When averaged over the six seasons, the *M6PR* transgene had no significant effect on development (Figure 2c). However, in two of the three fall seasons, M6PR plants had significantly earlier time to first flower (stage 5) by an average of 4.4 and 5.6 days (*p* value, orthogonal contrast of M6PR plants versus WT = .022 and .016, respectively) and time to 75% bolting (stage 6), by an average of 4.0 and 6.6 days (*p* value, orthogonal contrast of M6PR plants versus WT = .040 and .004, respectively). In the other seasons, the M6PR plants bolted and flowered an average of 0.4–2.3 days sooner. Seed viability, as measured by percent germination, did not differ significantly between the transgenic and parental lines for three transgenes (Table 2, Figure S4).

**Table 2:** Transgene effect on productivity as observed in pure line populations averaged across all field seasons

Line	Genotype	Dry weight (g)^a^	Seed yield (g)^a^	Percent germination^a^	Viable seed yield (g)^a^	Relative fitness (g viable seed transgenic/WT)
WS	WT	16.36	3.26	0.923	3.10	
A30	CBF3	10.68*	1.02*,^b^	0.870	1.32**	0.47*,^c^^,^ d
A40	CBF3	12.36	2.19*	0.924	2.12*	0.64*
Col(gl)	WT	13.91	1.81	0.933	1.69	
S1‐1	SOS1	12.54	2.10	0.919	2.03	1.20
S7‐6	SOS1	13.51	1.70	0.950	1.62	1.03
Col	WT	13.71	1.82	0.947	1.75	
M2‐1	M6PR	13.18	2.06	0.933	1.96	1.33
M5‐1	M6PR	11.17	2.10	0.945	2.00	1.23

a Each value is the mean of six seasons with five replicate populations per genotype per season.

b *,**,Value is significantly different from WT within transgene group, paired *t* test (by season) (*df* = 5), *p* < .05, 0.01, respectively.

c Each value is the mean of fitness estimates calculated for each of the six seasons. Each fitness estimate within a season was calculated as the mean viable seed yield of five replicate transgenic populations/mean seed yield of five replicate WT populations for that season.

d Mean fitness is significantly different from WT (*t* test, *n* = 6, H_0_: relative fitness =1).

The *CBF3* lines exhibited reduced dry weight, average seed yield, and viable seed yield, relative to the WT WS background (Table 2). *CBF3* plants also under‐performed relative to mean seed yield for the season (averaged over all WT and transgenic lines), except under the least favorable (i.e., lowest yielding) environments (Figure 1). Analysis of variance of seed yield for *CBF3* lines and their WT parent indicated highly significant (*p* < .001) season and season X genotype interactions (Table S3A). Examination of individual seasons by orthogonal contrast shows significantly reduced yield in the *CBF3* lines in four of the six seasons; there was not a significant effect of the *CBF3* gene in the two lowest yielding seasons (seasons 2 and 5).

In contrast, overexpression of *SOS1* and *M6PR* did not significantly impact productivity measures. Seed yield of the *SOS1* and *M6PR* transgenic lines largely paralleled average yield for each season and there did not appear to be a yield cost to the expression of *SOS1* and *M6PR* in high yielding environments under the conditions encountered in these experiments (Figure 1). While there were highly significant season effects on seed yield of the *SOS1* and *M6PR* lines and their respective WT parents (*p* < .001; Table S3B,C), there were not significant transgene or season X transgene interactions for seed yield.

The reduced viable seed yield of *CBF3* lines resulted in reduced fitness relative to WT WS (Table 2). Although the relative fitness of the *M6PR* lines averaged over the six seasons was 1.33 and 1.23 (Table 2), there was variation among the seasons. For example, while yield was greatly reduced for both parents and M6PR lines in the highly unfavorable seasons 2 and 5, the *M6PR* lines were less badly affected than the WT parent in the cool season 2, producing approximately twice as much seed. However, in the very wet season 5 the situation was reversed. Consistent with these observations, analysis of variance indicated a significant effect of season on fitness values for *M6PR* (Table S4C; *p* < .05). There was not significant variation among seasons for fitness effects of *CBF3* or *SOS1* (Table S4A,B).

### 3.2 Performance in competition with WT

Each of the transgenic lines was also grown in competition with their WT parents. Fourteen replicate populations of each line were initiated with a transgene frequency of 50% and maintained separately over six generations with a constant starting seeding density at each generation of ~2600 seeds/m^2^. Transgene frequencies were determined for each of the 14 populations for each transgene line at each generation by kanamycin screening. To rule out the possibility that apparent reduction in transgene frequency measured by kanamycin screening was an artifact due to gene silencing (i.e., the gene was present but not expressed), qPCR analysis was performed on progeny seed from all populations with frequency estimates below 0.15 after the sixth generation. The qPCR results verified that reduction in gene frequency observed from kanamycin screening resulted from reduced frequency of the transgene and not from gene silencing (Table S5).

**Figure 3:**
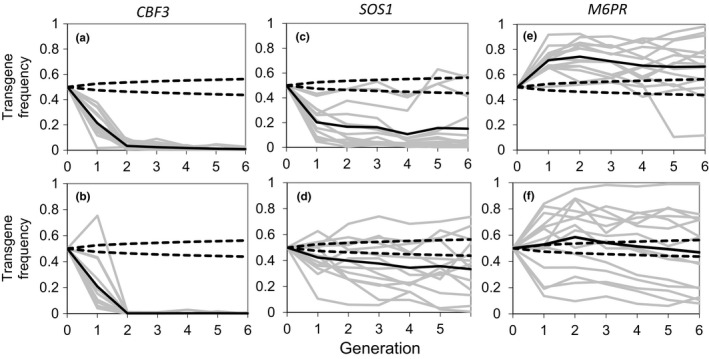
Transgene frequency within mixed populations. (a, b) wild‐type (WT) WS and *CBF3* overexpression lines, A40 (a) and A30 (b); (c, d) WT Col(gl) and *SOS1* lines, S1‐1 (c) and S7‐6 (d); and (e, f) WT Col and *M6PR* lines, M2‐1 (e) and M5‐1 (f), as determined by selectable marker screening for growth on kanamycin. Each value for each population is the mean of three replicate kanamycin screening plates/generation with 100 seedlings/plate. All populations began at 50% starting frequency (FG0) and were maintained separately in subsequent generations. Gray lines—transgene frequency in each of the 14 replicate mixed populations; solid black line—mean of the 14 replicate populations. Dashed lines indicate the 95% confidence intervals for populations undergoing solely genetic drift. Reductions in transgene frequency were confirmed by qPCR analysis at generation 6 (i.e., were not an artifact due to gene silencing) (Table S3)

Diverse establishment trends were observed among transgenes and independent lines. In competition with WT, *CBF3* plants were quickly driven to near‐extinction by the second generation in all populations (Figure 3a,b). The slow growing *CBF3* plants were frequently overgrown by neighboring WT plants that bolted and flowered sooner. In some cases, this further delayed development of the *CBF3* plants or prevented completion of their life cycle. Decreases in transgene frequency were also observed for many of the *SOS1* populations (Figure 3c,d). Mean values fell below the 95% confidence interval predicted from theoretical drift distributions (Falconer & Mackay, 1996), indicating negative selection against the transgenic plants. In contrast, populations of *M6PR* line M2‐1 exhibited a high mean transgene frequency, indicative of positive selection (Figure 3e,f). Individual populations of *M6PR* line M5‐1 diverged after the first generation and continued to separate in subsequent generations. In some populations, presence of the *M6PR* gene either reached near fixation or near elimination. Despite divergence among the populations, the mean value for the 14 replicates fell within the 95% confidence interval for wild‐type fitness, indicating that genetic drift was driving transgene frequency, as might be expected for small populations in the absence of selection.

**Figure 4:**
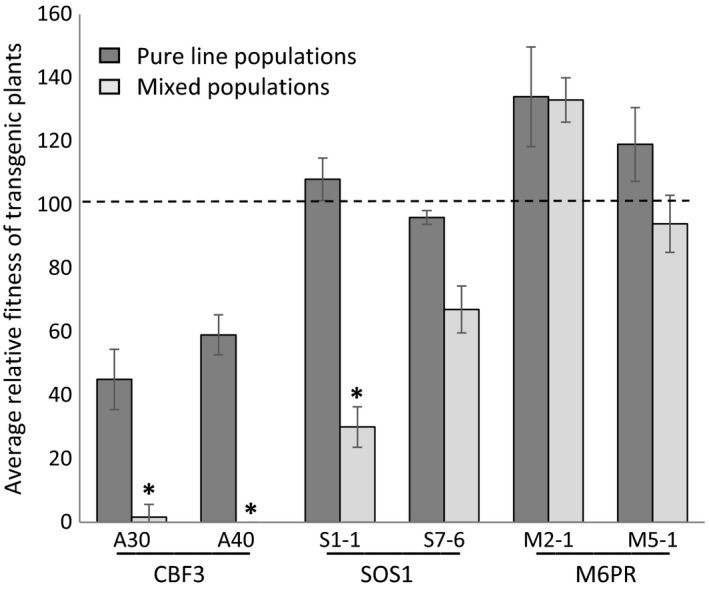
Comparison of fitness estimates for the three abiotic stress tolerance enhancing transgenes when estimated from pure line populations or in competition with WT parents. A fitness value of 100% (dotted line) indicates fitness equal to wild type. Each pure line fitness value is the mean transgene fitness ±SE., calculated from transgenic population seed yields relative to WT seed yields, of five replicate pure populations per generation, averaged across the six field seasons. Each mixed population fitness value is the mean transgene fitness ±SE, calculated by selectable marker screening of 14 replicate populations (three replicate kanamycin screening plates per population; 100 seeds per plate) at the end of six generations (i.e., each of the values reflects cumulative effects of the six seasons). Each population began as a 1:1 WT:transgenic mix and was maintained separately for the six generations; relative fitness is calculated with respect to expected transgene frequency of 0.5. * Pure line and mixed population fitness values were significantly different (Student's *t* test, *df *= 18, *p* < .05)

The proportion of transgenic seed in the mixed populations provides an estimate of relative fitness (i.e., observed transgene frequency relative to expected frequency of 50%, if fitness were equal) that can be compared to that obtained from fitness estimates of pure populations. Comparison of fitness estimates from mixed populations at the end of generation 6 to the mean of fitness estimates obtained from pure populations over the six generations, showed exacerbation of negative fitness effects when in competition with WT parents (Figure 4). Although *CBF3* lines showed decreased fitness in pure stands relative to WT WS, fitness in competition with WT was even more reduced. Fitness of *SOS1* line 1‐1 was comparable to WT based on pure populations, but when in competition with WT, it was significantly lower. *M6PR* estimates were not significantly different between pure line and mixed population estimates.

## 4 DISCUSSION

Mounting challenges due to climate change and global population growth will necessitate development of crops with increased resistance to abiotic stresses. While transgenic approaches may contribute to more resilient crops, deployment of transgenic crops is met with concerns about potential environmental impacts. Studies showing relationship between variation in key genes conferring stress‐related traits and geographic distribution suggest that such genes play a key role in adaptation to adverse environments (e.g., Baxter et al., 2010; Busoms et al., 2015). Thus, introgression of genes enhancing stress tolerance could provide a fitness advantage allowing for increased weediness or invasiveness of recipient populations in cases where the environmental stress is a limiting factor. On the other hand, adaptations favoring success in the face of abiotic and biotic stresses are frequently accompanied by reduced growth rate, leading to trade‐offs between competitiveness and survival that could influence potential for transgene establishment in native populations (Anderson, Willis, & Mitchell‐Olds, 2011; Claeys & Inze, 2013; Karabourniotis, Liakopoulos, Nikolopoulos, Bresta, & Sumbele, 2014; Trontin, Tisne, Bach, & Loudet, 2011). The objective of this study was to examine the fitness effects and persistence of three mechanistically diverse abiotic stress tolerance transgenes as observed over multiple generations and environmental conditions.

### 4.1 Potential for pleiotropy and genotype X environment interaction varies with the transgene

Multiple fitness components, such as growth rate, survival, and reproduction, as influenced by variable environmental conditions, ultimately drive individual and population success (Laughlin & Messier, 2015; Voille et al., 2007). While our APHIS field release permits did not allow us to test fitness effects at all stages of development in field conditions (e.g., germination, silique dehiscence, and seed dispersal), during their time in the field over the six seasons, the plants were exposed to a range of conditions that markedly influenced both vegetative and reproductive productivity. Fitness estimates were calculated from grams of viable seed produced per population, as assessed from total seed weight and percent germination measured for each population at each generation. Relative seed production in comparison with WT comparators varied among the transgenes.

Pure populations of *SOS1* and *M6PR* plants exhibited generally neutral or positive fitness effects. These effects are consistent with growth chamber trials showing comparable performance to WT when grown in the absence of salt stress (Chan et al., 2012). In contrast, *CBF3* plants exhibited markedly reduced fitness. The negative fitness effect of *CBF3* is likely due to an associated dwarfing phenotype, as has been previously observed in growth chamber and glasshouse studies (Gilmour et al., 2000; Jackson, Stinchcombe, Korves, & Schmitt, 2004), and delayed development as was observed in these field experiments. Inferred negative fitness effects of *CBF* genes in climates that are not subject to extensive cold stress also have been observed in naturally occurring *Arabidopsis* populations. Populations from warmer climates exhibited reduced induction of *CBF* regulon genes in response to cold, and occurrence of nonsynonymous or frame‐shift alleles influencing functionality of the *CBF* genes was observed in association with populations from warmer climates (Gehan et al., 2015; Monroe et al., 2016; Oakley, Agren, Atchison, & Schemske, 2014).

Although the plants experienced a range of growing conditions, the abiotic stresses for which these genes have been previously shown to improve tolerance in the growth chamber were not specifically applied [e.g., salinity stress for *SOS1*,* CBF3,* and *M6PR;* freezing and drought for *CBF3* (Gilmour et al., 2000; Kasuga, Liu, Miura, Yamaguchi‐Shinozaki, & Shinozaki, 1999; Shi et al., 2003; Zhifang & Loescher, 2003)]. Thus, the observed transgene effects, both positive and negative, indicate pleiotropic effects and GxE interactions. Given the function of the introduced transgene products (transcription factor, sodium antiporter, compatible solute and possible pathogen signal molecule) and thus their capacity to modify a range of cellular functions, their extensive influence on the transcriptome (Chan et al., 2012), and the known interplay among plant responses to multiple abiotic and biotic stresses (Krasensky & Jonak, 2012; Mittler and Blumwald 2010), it was anticipated that the transgenes would cause pleiotropic effects, but that the extent of pleiotropy could vary with the transgene, as was observed in these experiments. Of course, the fitness effects observed with the *CBF3* lines in the higher yielding environments in these experiments were quite extreme; cultivars aimed at increasing crop production would not be anticipated to have strong negative effects on productivity.

### 4.2 Relative performance is influenced by intergenotypic competition

Processes of transgene introgression and establishment in native populations will require successful competition with plants within the population that do not possess the transgene. In the case of a crop and its interfertile wild relatives, a range of differences may impact fitness of the resulting hybrids, including some, such as domestication traits, that may have negative effects on the progeny (Campbell et al., 2009; Hails & Morley, 2005; Hooftman et al., 2011; Lu et al., 2016). The intent of these experiments was examine the effect of the transgene per se, isolated from other potentially confounding background genetic differences. Therefore, the transgenic lines were planted in direct competition with their respective WT parents. The seed planted for each generation was a subset of the progeny seed produced in the previous generation, allowing the impacts of one season and transgene combination to influence the transgene frequency in the next season. This method follows the natural processes of selection and drift that would occur during establishment of a new genotype in the environment. The three transgenes followed different trajectories over time. When in competition with WT parental lines, the negative effects of *CBF3* were intensified leading to transgene extinction. The *SOS1* 1‐1 plants, which had shown equivalent fitness in pure populations, instead displayed impaired fitness when in competition with WT. *M6PR* M2‐1 showed an average increase in transgene frequency while populations of M5‐1 changed in a stochastic fashion overtime, implicating genetic drift.

The few studies that have compared effects of transgenes in the presence and absence of intergenotypic competition, also have shown differences in fitness estimates from the two methods. For example, insect‐resistant (Bt) rice exceeded yield of WT under insect pressure in pure populations, but lost that advantage in competition (Yang, Wang, Su, & Lu, 2012). Transgenic insect‐resistant canola produced more seed in pure populations than WT, but did not show increased seed yield at all sites when grown in 1:1 competitive populations with WT canola (Ramachandran, Buntin, All, Raymer, & Stewart, 2000). An inverse effect was observed for transgenic rice expressing a Bt gene for insect resistance (Liu, Ge, Liang, Wu, & Li, 2015). In the absence of competition with WT, the transgenic rice did not produce as much seed as WT, both in the absence and presence of insect pressure; however, in most cases, the disadvantage was reduced or lost when planted in mixed populations with WT. While the above competition studies were replicated in locations and/or years, to our knowledge, this is the first study that provides longitudinal data following populations over multiple generations.

In addition to direct differences resulting from intergenotypic competition versus performance in pure lines, the estimates obtained from the mixed populations in this study reflect cumulative effects of the fitness impacts in the prior generation. Thus, as would occur during introgression into native populations, the effects of relative fitness differences would be compounded from one generation to the next. Depending on the nature of the genetic differences and selective environment, intraspecific competition can result in additive, antagonistic, or neutral effects (Weis & Hochberg, 2000). In the case of additive competition, a fitness disadvantage is amplified by increased competition from more rapidly growing plants. This appears to be the case in the intergenotype populations with *CBF3* plants, which were overgrown and inhibited from reaching full development by their WT neighbors, resulting in more rapid decline in representation in the population than was predicted based on the pure populations. However, despite somewhat earlier flowering for the *M6PR* plants, there did not appear to be effects of additive competition in intergenotypic populations under the conditions tested in these experiments. This is likely due to the relatively modest differences in rate of development. The *M6PR* plants did not exhibit increased vegetative growth as measured by aboveground dry weight at harvest, and so likely did not cause increased competition for resources such as light, water, and nutrients that can contribute to additive competitive effects. In several cases (*CBF3* A30 and A40, *SOS1* 1‐1 and *M6PR* 2‐1), there was a marked effect of the transgene in the first generation that was subsequently maintained or accelerated for most populations in the following generations. While *CBF3* lines, which exhibited consistent negative fitness regardless of season, would likely exhibit a rapid decline under almost all conditions, it is possible that different trajectories would have been observed had the *SOS1* or *M6PR* populations been initiated in different seasons with different selective pressures.

Clearly competition experienced by plants within natural settings will not be limited to intraspecific competition. Plants encounter competition from other plant species as well as biotic stresses such as herbivory (Mercer et al., 2014; Weis & Hochberg, 2000). While it is not possible to account for all possible factors, a few studies directly comparing inter‐ and intraspecific competition have found that intraspecific competition, and especially population density, plays a predominant role in relative fitness. For example, although *Brassica rapa* and *B. napus* both suffered when in competition with *Lolium perenne*, plant density, regardless of species, had a greater impact on performance, even in the presence of insect herbivore pressure (Damgaard & Kjaer, 2009). Similarly, conspecific plant density had a greater influence on fitness parameters of sunflower and crop‐wild hybrids than did the presence of interspecific competition from other weed species (Mercer et al., 2014).

## 5 CONCLUSION

A series of multiyear, multigenerational experiments found that three mechanistically different abiotic stress resistance transgenes exhibited different fitness effects in *Arabidopsis thaliana,* likely reflecting diverse effects on gene expression, plant development, and productivity when exposed to varying environmental conditions. Thus potential impacts following transgene release are likely to be highly dependent on the specific nature of the transgene and its physiological effects, necessitating case‐by‐case evaluation. Fitness estimates also differed between pure populations and intergenotype competitive populations. Negative fitness effects were heightened in competition and magnified as populations were carried forward over generations. These findings demonstrate the importance of competition in influencing transgene persistence and illustrate the compounding effects of relative fitness effects over generations as would occur during gene introgression. These multigenerational studies indicate that competitive field trials could aid evaluation of genetically engineered crops in cases where there are concerns over transgene establishment in noncrop populations.

## CONFLICT OF INTEREST

None declared.

## Supporting information


** **
