# Supplementary material for: Influence of intergenotypic competition on multigenerational persistence of abiotic stress resistance transgenes in populations of Arabidopsis thaliana
Source: Evol Appl. 2018 Mar 5;11(6):950–62. doi: 10.1111/eva.12610 (PMC5999209; doi:10.1111/eva.12610)
Supplement: Supplementary file 1 [file EVA-11-950-s001.docx]

| **Supplementary Table 1.** Transgenic *Arabidopsis thaliana* lines engineered with abiotic stress tolerance genes and the number of replicate populations tested in each of the six generations in the field. | | | | | | |
| --- | --- | --- | --- | --- | --- | --- |
| **Wild-type**^1^ | | | **Transgene** | **Transgenic line** | **Number of Reps** | **Initial Mix Ratio** |
| WS | | |  |  | 5 | Unmixed |
| Col | | |  |  | 5 | Unmixed |
| Col(gl) | | |  |  | 5 | Unmixed |
|  | | | CBF3 | A30 | 5 | Unmixed |
|  | | | CBF3 | A40 | 5 | Unmixed |
|  | | | SOS1 | S1-1 | 5 | Unmixed |
|  | | | SOS1 | S7-6 | 5 | Unmixed |
|  | | | M6PR | M2-1 | 5 | Unmixed |
|  | | | M6PR | M5-1 | 5 | Unmixed |
| WS | | | CBF3 | A30 | 14 | 1:1 |
| WS | | | CBF3 | A40 | 14 | 1:1 |
| Col(gl) | | | SOS1 | S1-1 | 14 | 1:1 |
| Col(gl) | | | SOS1 | S7-6 | 14 | 1:1 |
| Col | | | M6PR | M2-1 | 14 | 1:1 |
| Col |  |  | M6PR | M5-1 | 14 | 1:1 |

^1^Wild-types are Wassilewskija (WS), Columbia glabrous (Col(gl)), and Columbia (Col) ecotypes

| **Supplementary Table 2.** Seasonal effect on development and productivity averaged across all lines and populations**.** | | | | | | | | | | | | | | | |
| --- | --- | --- | --- | --- | --- | --- | --- | --- | --- | --- | --- | --- | --- | --- | --- |
|  |  |  | Days to reach Lifecycle Stage | | | | | | | |  | Productivity | |  | |
| Field Genera-tion | Time in field | Season | Germi-nation | 2 True Leaves | 5-6 True Leaves | First Bolting | Majority Bolting  (75%) | First Flower-ing | Majority Flowering  (75%) | Mature  (75% dry siliques) |  | Dry Weight (g) | Seed Yield (g) | |  |
| 1 | June -July 08 | Summer | 4.31c^1^ | 12.13b | 16.25a | 20.18a | NA | 23.58a | NA | 44.71a |  | 12.33b | 1.33d | |  |
| 2 | Sept - Nov 08 | Fall | 7.82d | 17.51d | 28.02c | 37.31b | 55.64c | 46.02c | 67.52d | 118.8e |  | 10.6bc | 0.91e | |  |
| 3 | May - June 09 | Spring | 4.02c | 12.9c | 19.06b | 20.74a | 27ab | 24.66a | 32.14ab | 53.18b |  | 17.36a | 3.56a | |  |
| 4 | Sept - Oct 09 | Fall | 3.06a | 11.58a | 18.88b | 20.96a | 25.22a | 26.22a | 30.98a | 55.0b |  | 9.95c | 2.34c | |  |
| 5 | May - June 10 | Spring | 4.0c | 12.38c | 18.64b | 22.95a | 28.85b | 32.74b | 37.47c | 57.83c |  | 7.45d | 0.83e | |  |
| 6 | Sept - Oct 10 | Fall | 3.48b | 12.91c | 18.5b | 19.74a | 27.48ab | 27.53a | 34.66b | 68.25d |  | 18.72a | 2.72b | |  |
| ^1^Each value is the mean of all pure-line populations grown during a season (n=45). Means with the same letter are not significantly different from each other at P<0.05 (Analysis of variance, Duncan’s multiple range test). Measures of productivity were square root transformed prior to analysis of variance. Values shown are untransformed for clarity. | | | | | | | | | | | | | | | |

| **Supplementary Table 3A**. Analysis of variance of seed yield of pure populations of WS and *CBF3* A30 and A40 grown in six field seasons. | | | | | |
| --- | --- | --- | --- | --- | --- |
| Source | Degrees of freedom | Sum of Squares | Mean Square | F Value | Probability |
| Model | 17 | 172.67 | 10.16 | 14.60 | <.001 |
| Environment (season) | 5 | 19.28 | 3.86 | 5.54 | <.001 |
| Genotype | 2 | 61.60 | 30.80 | 44.25 | <.001 |
| Genotype*Environment | 10 | 91.79 | 9.18 | 13.19 | <.001 |
| WS vs. CBF season 1 | 1 | 6.98 | 6.98 | 6.98 | .011 |
| WS vs. CBF season 2 | 1 | 0.37 | 0.37 | 0.37 | ns |
| WS vs. CBF season 3 | 1 | 40.17 | 40.17 | 40.17 | <.001 |
| WS vs. CBF season 4 | 1 | 21.36 | 21.36 | 21.36 | <.001 |
| WS vs. CBF season 5 | 1 | 2.63 | 2.63 | 2.63 | ns |
| WS vs. CBF season 6 | 1 | 32.73 | 32.73 | 32.73 | <.001 |
| Error | 72 | 50.13 | 0.87 | 0.70 |  |
|  | | | | | |

| **Supplementary Table 3B**. Analysis of variance of seed yield of pure populations of Col(gl) and *SOS1* 1-1 and 7-6 grown in six field seasons. | | | | | |
| --- | --- | --- | --- | --- | --- |
| Source | Degrees of freedom | Sum of Squares | Mean Square | F Value | Probability |
| Model | 17 | 128.17 | 7.54 | 14.04 | <.001 |
| Environment (season) | 5 | 115.61 | 23.12 | 43.06 | <.001 |
| Genotype | 2 | 2.65 | 1.32 | 2.47 | ns |
| Genotype*Environment | 10 | 9.92 | 0.99 | 1.85 | ns |
| Error | 72 | 38.64 | .537 |  |  |
|  | | | | | |

| **Supplementary Table 3C**. Analysis of variance of seed yield of pure populations of Col and *M6PR* 2-1 and 5-1 grown in six field seasons. | | | | | |
| --- | --- | --- | --- | --- | --- |
| Source | Degrees of freedom | Sum of Squares | Mean Square | F Value | Probability |
| Model | 17 | 135.39 | 7.96 | 6.40 | <.001 |
| Environment (season) | 5 | 118.23 | 23.64 | 19.07 | <.001 |
| Genotype | 2 | 1.33 | 0.66 | 0.53 | ns |
| Genotype*Environment | 10 | 15.83 | 1.58 | 1.28 | ns |
| Error | 72 | 89.49 |  |  |  |
|  | | | | | |

| **Supplementary Table 4A**. Analysis of variance of relative fitness (relative seed yield of pure populations) of *CBF3* A30 and A40 vs WT WS grown in six field seasons. | | | | | |
| --- | --- | --- | --- | --- | --- |
| Source | Degrees of freedom | Sum of Squares | Mean Square | F Value | Probability |
| Model | 6 | 1.046 | 0.174 | 2.231 | ns |
| Environment (season) | 5 | 0.947 | 0.189 | 2.417 | ns |
| Line | 1 | 0.099 | 0.099 | 1.269 | ns |
| Error | 5 | 0.391 | 0.078 |  |  |
| **Supplementary Table 4B**. Analysis of variance of relative fitness (relative seed yield of pure populations) of pure populations of *SOS1* 1-1 and 7-6 vs WT Col(gl)] grown in six field seasons. | | | | | |
| Source | Degrees of freedom | Sum of Squares | Mean Square | F Value | Probability |
| Model | 6 | 0.428 | 0.071 | 0.973 | ns |
| Environment (season) | 5 | 0.382 | 0.076 | 1.041 | ns |
| Line | 1 | 0.046 | 0.046 | 0.631 | ns |
| Error | 5 | 0.367 | 0.073 |  |  |
| **Supplementary Table 4C**. Analysis of variance of relative fitness (relative seed yield of pure populations) of pure populations of *M6PR* 2-1 and 5-1 vs WT Col grown in six field seasons. | | | | | |
| Source | Degrees of freedom | Sum of Squares | Mean Square | F Value | Probability |
| Model | 6 | 5.001 | 0.834 | 5.209 | 0.047 |
| Environment (season) | 5 | 4.935 | 0.987 | 6.176 | 0.037 |
| Line | 1 | 0.066 | 0.066 | 0.413 | ns |
| Error | 5 | 0.799 | 0.160 |  |  |

| **Supplementary Table 5.** Comparison of transgene frequencies estimated by phenotypic selectable marker screening (nptII) and qPCR analysis. | | | |
| --- | --- | --- | --- |
| **Transgene** | **Sampled populations^1^** | **Estimated transgene frequency** | |
|  |  | **nptII screening^2^** | **qPCR analysis^3^** |
| M6PR | Positive control | 100.0 | >90.0 |
| None (WT) | Negative control | 0.0 | <DL^4^ |
| CBF3 | A30 #7 | 0.007 | <DL |
| CBF3 | A40 #2 | 0.027 | <DL |
| SOS1 | S1-1 #6 | 0.003 | <DL |
| SOS1 | S1-1 #11 | 0.0 | <DL |
| SOS1 | S1-1 #14 | 0.003 | <DL |
| SOS1 | S7-6 #1 | 0.007 | <DL |
| SOS1 | S7-6 #5 | 0.05 | <DL |
| SOS1 | S7-6 #6 | 0.133 | <0.1 |
| M6PR | M2-1 #14 | 0.117 | <0.1 |
| M6PR | M5-1 #3 | 0.08 | <0.1 |
| M6PR | M5-1 #8 | 0.127 | <0.1 |
| M6PR | M5-1 #14 | 0.107 | <0.1 |

^1^All sixth generation competitive populations estimated to have < 15% transgenic individuals by phenotypic selectable marker (nptII) screening were tested by qPCR.

^2^Each value is the mean frequency estimated from three replicate screenings of 100 seedlings each.

^3^Each value is the mean frequency from three replicate biological samples with four technical replicates per biological sample. Frequency estimates were calculated based on a standard qPCR CT curve with mixes containing 0%, 10%, 20%, 50% and 100% transgenic seedlings.

^4^Frequency below detection limits (DL) of 0.1.


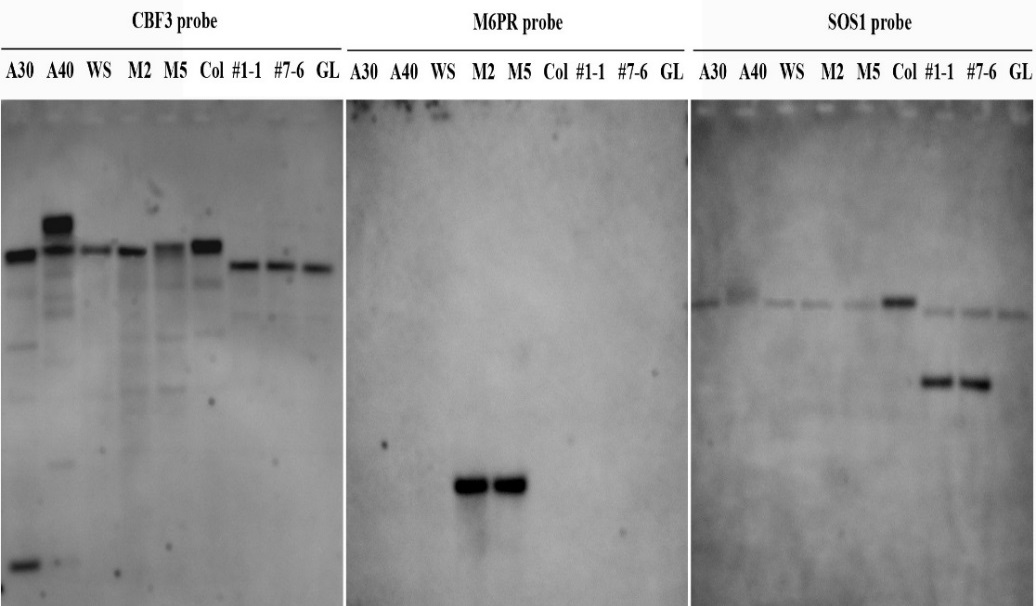


Probes

Arabidopsis lines


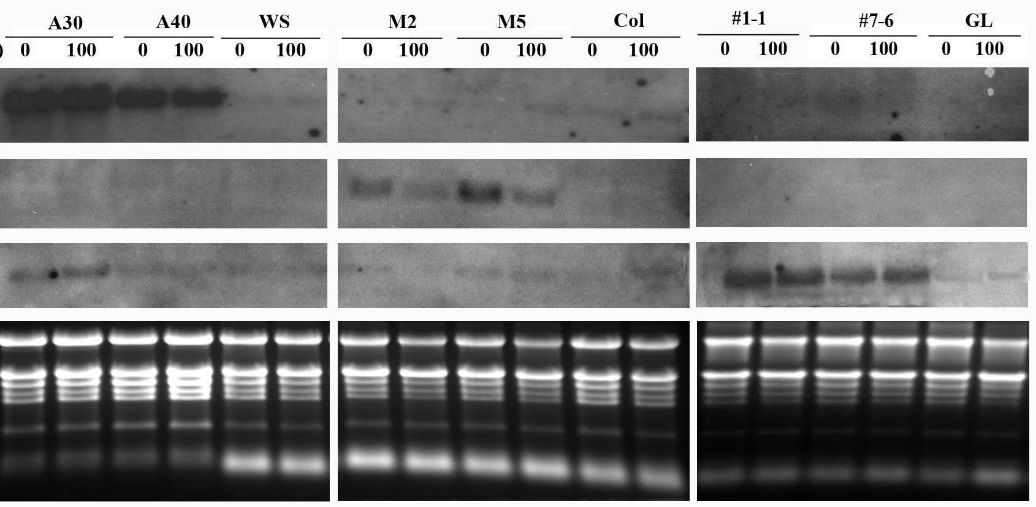


Salt concentration (mM)

Arabidopsis lines

*CBF3* probe

*M6PR* probe

*SOS1* probe

RNA

**a**

**b**

**Supplementary Figure 1. Verification of transgenic Arabidopsis thaliana lines via Southern (a) and northern analysis (b).** SOS1 and CBF3 lines show the respective transgene and the endogenous gene, while M6PR lines show only the transgene since the gene is not endogenous to *Arabidopsis thaliana.*


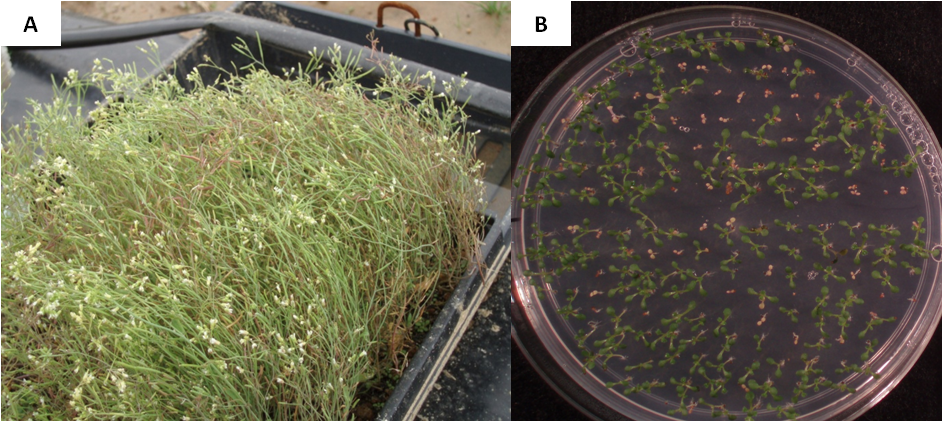


**Supplementary Figure 2. The field planting method and selectable marker screening of progeny seed.** (A) The tray-in-flat field planting method showing the trickle hose for irrigation and the high density of planting for interplant competition. A single planted 26x26 cm tray was placed in the center of a 27.4x53.9 flat, the larger flat was anchored to the ground using 15.2 cm landscaping stakes, to prevent movement or tipping of the planted trays. (B) Screening of progeny seed on ½ MS 1% agar 100mg/L kanamycin containing media. Individual seeds or seedlings are characterized as: wild-type (bleached - examples indicated by white arrows), transgenic (green) or un-germinated (red arrow).

**B**

**A**

**Supplementary Figure 3. Seasonal differences in maximum and minimum air temperature (a) and daily total solar flux density (b) across the six field generations.** Each value is the mean ± S.E.M. across all days populations were in the field for that growing season.


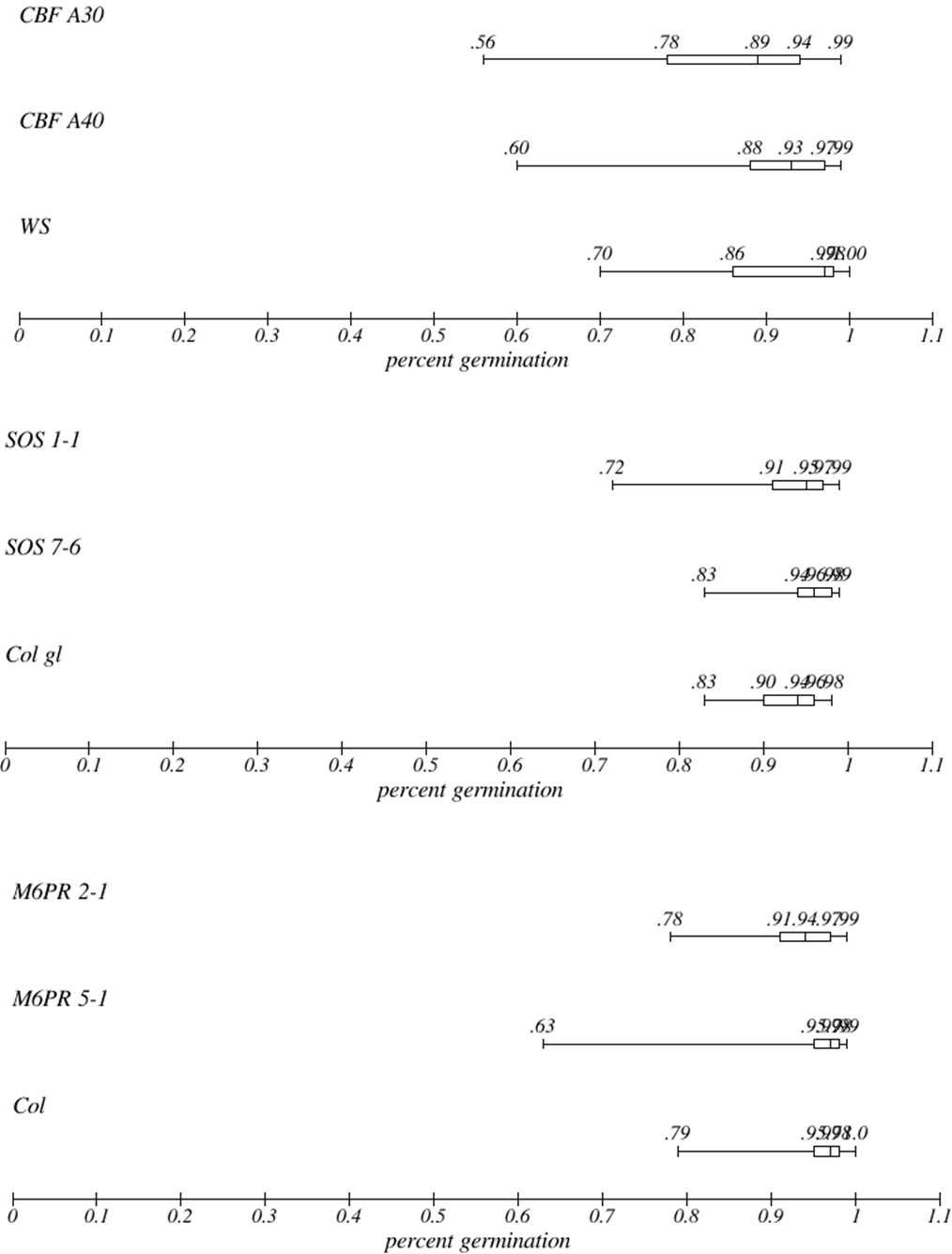


**Supplementary Figure 4.** Box plots of percent germination for seed from pure line populations. (n=30; 6 seasons, 5 replicate populations/season).
